# Association of Inflammatory and Metabolic Markers with Mortality in Patients with Postoperative Femur Fractures in the Intensive Care Unit

**DOI:** 10.3390/medicina61030538

**Published:** 2025-03-19

**Authors:** Metin Kilinc, Enes Çelik, Ibrahim Demir, Semih Aydemir, Hakan Akelma

**Affiliations:** 1 Department of Anesthesiology and Reanimation, Faculty of Medicine, Mardin Artuklu University, Mardin 47200, Turkey; anestezistenescelik@gmail.com (E.Ç.); hakanakelma@hotmail.com (H.A.); 2 Department of Anesthesiology and Reanimation, Mardin Training and Research Hospital, Mardin 47200, Turkey; dribo21@hotmail.com; 3 Department of Anesthesiology and Reanimation, Yenimahalle Training and Research Hospital, University of Yıldırım Beyazit, Ankara 06370, Turkey; drsemihaydemir@gmail.com

**Keywords:** postoperative femur fracture, ICU mortality, inflammatory markers, APACHE II score, albumin, CRP, Pan-Immune-Inflammation Value, neutrophil-to-lymphocyte ratio

## Abstract

*Background and Objectives*: Postoperative femur fracture in elderly patients is associated with high morbidity and mortality, especially in the intensive care unit (ICU). Various factors, including demographic and laboratory parameters, may influence mortality in this population. The aim of this study was to evaluate the association of inflammatory and metabolic markers with mortality in ICU patients with postoperative femur fractures and to identify key predictors to enhance risk stratification and improve patient outcomes. *Materials and Methods*: In this retrospective single-center study, we analyzed 121 patients aged over 65 years with postoperative femur fractures who were admitted to the ICU between January 2023 and January 2024. Demographic and clinical data, including comorbidities, Charlson Comorbidity Index (CCI), and Acute Physiology and Chronic Health Evaluation (APACHE II) score, were collected. Laboratory parameters such as white blood cell count (WBC), albumin, C-reactive protein (CRP), D-dimer, Pan-Immune-Inflammation Value (PIV), CRP-to-albumin ratio (CAR), neutrophil-to-lymphocyte ratio (NLR), and others were analyzed. Linear regression, logistic regression, and Receiver Operating Characteristic (ROC) analyses were performed to determine the predictive value of these markers for ICU mortality. *Results*: The mean age of the patients was 76.3 ± 9.6 years, and 52.1% were female. The most common comorbidities were hypertension (67.8%) and diabetes (49.6%). ICU mortality occurred in 24 patients (20%). Significant predictors of mortality included higher CRP (>62.8 mg/L), NLR (>10.0), PIV (>450), and APACHE II scores (>23) (*p* < 0.001 for all). Lower albumin levels (<2.5 g/dL) were strongly associated with increased mortality (*p* < 0.001). ROC analysis demonstrated that the APACHE II score had the highest predictive accuracy for mortality (AUC = 0.83), followed by albumin (AUC = 0.79) and PIV (AUC = 0.76). Extended ICU stay (>10 days) was also significantly correlated with increased mortality (*p* < 0.001). *Conclusions*: This study successfully demonstrates the utility of combining traditional clinical markers, such as APACHE II score, with novel inflammatory markers, such as PIV, CAR, and NLR, in predicting mortality in ICU patients following femur fracture surgery. The integration of emerging biomarkers with well-established scoring systems offers enhanced predictive accuracy and provides valuable insights into patient management.

## 1. Introduction

Femur fractures are a significant health concern, particularly in the geriatric population, and are associated with high morbidity and mortality rates [1,2]. Surgical intervention is often required, and postoperative complications frequently contribute to adverse outcomes, especially in patients admitted to intensive care units (ICUs) [1,2]. Geriatric patients are at increased risk due to multiple comorbidities, limited physiological reserves, and heightened susceptibility to systemic inflammation and metabolic disturbances following trauma [3,4]. Recent studies have highlighted that biomarkers such as C-reactive protein (CRP) and albumin levels can provide early prognostic insights into postoperative outcomes in geriatric patients with femur fractures [5,6,7].

Traditional markers for predicting postoperative mortality include demographic factors such as age, gender, and comorbidities, as well as established clinical scoring systems like the APACHE II score and the Charlson Comorbidity Index (CCI) [5,6]. However, while these tools provide valuable insights into overall patient risk, they often fail to capture dynamic changes in inflammatory and metabolic status that may critically influence outcomes during the postoperative period.

Various demographic and laboratory parameters may have an association with mortality in patients with femur fractures. In addition to demographic factors such as age, gender, and comorbidities, biochemical parameters such as inflammatory response and metabolic status also stand out as determinants of postoperative outcomes [8,9]. In recent years, findings have been obtained suggesting that inflammatory markers such as neutrophil–lymphocyte ratio (NLR), CRP, procalcitonin, and platelet–lymphocyte ratio (PLR) may be strong indicators for predicting mortality in the postoperative period [10,11,12,13]. A meta-analysis further confirmed that elevated NLR and CRP levels significantly correlate with higher mortality risk in ICU patients with postoperative fractures [14,15]. These markers may provide important information about the clinical course by reflecting the body’s systemic inflammatory response to trauma.

In recent years, various biomarkers reflecting the inflammatory response and immune status have been used to predict postoperative complications and mortality. Pan-Immune-Inflammation Value (PIV) stands out as a new biomarker that evaluates the severity of the systemic inflammatory response by combining various components of the immune response (neutrophil, lymphocyte, and platelet counts) [16,17]. The CRP-to-albumin ratio (CAR) plays an important role in predicting postoperative outcomes in critically ill patients by evaluating inflammation and nutritional status together [18]. In particular, increased CRP levels and decreased albumin levels are closely associated with poor prognosis. Another important marker, the CRP-to-lymphocyte ratio (CLR), has been associated with the development of postoperative complications by reflecting the intensity of the inflammatory response [18]. High CLR values generally indicate the severity of systemic inflammation and are associated with the risk of mortality [19].

Existing biomarkers such as APACHE II and CCI, while robust, have limitations in reflecting the evolving inflammatory and immune responses seen in ICU patients following major surgery. This has driven the exploration of novel biomarkers like PIV, CAR, and CLR, which offer a more nuanced understanding of the interplay between inflammation, immune status, and metabolic health. By incorporating these emerging markers into routine risk assessment, clinicians may be better equipped to identify high-risk patients, personalize treatment approaches, and improve postoperative survival rates.

The aim of this study was to evaluate the association of inflammatory and metabolic markers with mortality in ICU patients with postoperative femur fractures and to identify key predictors to enhance risk stratification and improve patient outcomes.

## 2. Materials and Methods

### 2.1. Study Design and Study Population

This study was conducted in Mardin Training and Research Hospital, Turkey, to examine the demographic and laboratory data of patients who were admitted to the Mardin Training and Research Hospital Orthopedic Service due to femur fractures between 1 January 2023 and 1 January 2024 and who were admitted to the intensive care unit (ICU) in the postoperative period. The age, gender, comorbid diseases (diabetes mellitus, hypertension, coronary artery disease (CAD), heart failure, chronic kidney disease (CKD), chronic obstructive pulmonary disease (COPD), and malignancy), hospital stay (days), and ICU mortality status of the patients included in the study were recorded. In addition, the Charlson Comorbidity Index (CCI) and APACHE II scores of the patients were calculated and recorded.

Patients over the age of 65 who were admitted to the orthopedic service due to femur fracture in the preoperative period and were admitted to the intensive care unit in the postoperative period were included in the study. Patients under the age of 65, patients admitted from the emergency department, and patients with other traumas or pathologies other than isolated femur fracture were excluded from the study. Missing data were addressed through complete-case analysis. Patients with significant missing data for key variables were excluded, while those with minimal missing data (<5%) were handled using mean or median imputation.

### 2.2. Laboratory Parameters

Laboratory data of the patients, obtained at the time of ICU admission, were examined, and white blood cell (WBC) (×10^3^/µL), red cell distribution width (RDW) (%), platelet (Plt) (×10^3^/µL), neutrophil (×10^3^/µL), mean platelet volume (MPV) (fL), monocyte (×10^3^/µL), lymphocyte (×10^3^/µL), calcium (Ca++) (mg/dL), glucose (mg/dL), albumin (g/dL) (ALB), blood urea nitrogen (BUN) (mmol/L), creatinine (mg/dL), troponin (U/L), D-dimer (mg/L), C-reactive protein (CRP) (mg/L), and procalcitonin (ng/L) values were recorded. In addition, the following indices were calculated to estimate inflammatory and metabolic markers:

Pan-Immune-Inflammation Value (PIV): [(Neutrophil × Platelet)/Lymphocyte].
CRP-to-albumin ratio (CAR): CRP/Albumin.
CRP-to-lymphocyte ratio (CLR): CRP/Lymphocyte.
Platelet-to-lymphocyte ratio (PLR): Platelet/Lymphocyte.
Neutrophil-to-lymphocyte ratio (NLR): Neutrophil/Lymphocyte.

### 2.3. Statistical Analysis

Statistical analysis was performed using SPSS software, version 27.0 (SPSS Inc., Chicago, IL, USA). Continuous variables were expressed as the mean ± standard deviation, while categorical data were summarized as percentages. The Kolmogorov–Smirnov test was utilized to determine the normality of the data distribution. For data following a normal distribution, parametric tests were applied, whereas non-parametric methods were used for non-normally distributed data. The chi-square test was employed for the analysis of categorical variables, and the independent-samples *t*-test was used to compare means between two groups. Pearson correlation coefficients were calculated to examine the strength and direction of associations between continuous variables. Additionally, linear regression analysis was carried out to assess the impact of various parameters on mortality. Receiver Operating Characteristic (ROC) analysis was used to evaluate the diagnostic accuracy of biomarkers in predicting ICU mortality. The area under the ROC curve (AUC) reflects the overall performance of the test. Tests with an AUC value above 0.5 perform better than random guessing, with AUC values between 0.7 and 0.8 indicating moderate discrimination, values between 0.8 and 0.9 indicating good discrimination, and values above 0.9 representing excellent discriminatory power. Cut-off values were determined using the highest Youden Index (sensitivity + specificity − 1) during ROC analysis. This index identifies the point at which the true-positive rate is maximized while the false-positive rate is minimized. The cut-off values obtained were consistent with clinically significant mortality markers and aligned with previous studies in the literature [20,21]. Sensitivity, specificity, and positive and negative predictive values obtained from the ROC analysis are presented in table format, and the analysis results are visualized with a graph. A *p*-value of less than 0.05 was deemed statistically significant.

## 3. Results

Sociodemographic and laboratory findings of patients with postoperative femur fracture in the ICU are shown in Table 1. The mean age of the patients was 76.3 ± 9.6 years, and 52.1% were female. The most common comorbidities in the patients were hypertension (67.8%) and diabetes (49.6%). CAD was detected in 28.9% of the patients. When examined in terms of laboratory findings, the mean CRP level of the patients was 50 ± 20 mg/L, the albumin level was 3.5 ± 0.5 g/dL, and the d-dimer value was 1.5 ± 0.5 mg/L. The median value of PIV evaluating the inflammatory response was found to be 406.50 ± 102.50. In addition, NLR was calculated as 4.5 ± 1.8, while CAR was calculated as 1.5 ± 0.5. The mean APACHE II score was found to be 18 ± 5, and the mean duration of stay in the intensive care unit was determined to be 8.2 ± 5.6 days. A total of 24 patients (20%) in the study population died during the intensive care period (Table 1).

Demographic and laboratory parameter differences between survivors and non-survivors of postoperative femur fracture are outlined in Table 2. While the mean WBC value in the mortality group was 12.2 ± 3.7 × 10^3^/µL, this value was measured as 10.8 ± 3.2 × 10^3^/µL in surviving patients (*p* = 0.03). The RDW value determined as 16.3 ± 2.1% in the mortality group was significantly higher than in survivors (15.2 ± 1.9%) (*p* = 0.04). The mean lymphocyte level in patients in the mortality group was 1.3 ± 0.5 × 10^3^/µL, while it was found to be 1.6 ± 0.6 × 10^3^/µL in surviving patients (*p* = 0.04). Albumin levels were significantly lower in the mortality group (3.2 ± 0.6 g/dL), which was a significant difference from the albumin levels in surviving patients (3.7 ± 0.4 g/dL) (*p* = 0.001). The mean creatinine value was 1.2 ± 0.4 mg/dL in the mortality group, which was significantly higher than in survivors (*p* = 0.02). Troponin levels were also higher in the mortality group (*p* = 0.01). The CRP value was 70 ± 28 mg/L in the mortality group, while it was 48 ± 19 mg/L in survivors (*p* = 0.02). D-dimer levels were also higher in the mortality group (*p* = 0.001). CCI and APACHE II scores were also significantly higher in the mortality group (*p* = 0.04 and *p* = 0.001, respectively). In the mortality group, the PIV value was 470 ± 140, which was significantly higher than in the survivor group (350 ± 120) (*p* = 0.03). While the CAR value in the mortality group was 2.1 ± 0.8, it was found to be 1.4 ± 0.6 in the survivors (*p* = 0.02). Similarly, CLR was found to be significantly higher in the mortality group (*p* = 0.04) (Table 2).

The correlation between the length of hospital stay and mortality of patients with postoperative femur fracture in the ICU is shown in Table 3. Low albumin levels showed a negative correlation with both long hospital stays (r = −0.48, *p* < 0.001) and mortality (r = −0.52, *p* < 0.001). CRP (r = 0.49, *p* < 0.001) and procalcitonin (r = 0.46, *p* < 0.001) levels showed a positive correlation with both length of stay (LOS) and mortality. CCI and APACHE II scores showed a positive correlation with both LOS (r = 0.50, *p* < 0.001 and r = 0.54, *p* < 0.001) and mortality (r = 0.55, *p* < 0.001 and r = 0.60, *p* < 0.001). PIV (r = 0.41, *p* = 0.002 and r = 0.48, *p* = 0.001) and NLR (r = 0.48, *p* < 0.001 and r = 0.53, *p* < 0.001) were significant indicators reflecting the strength of the inflammatory response in both conditions and showed a significant positive correlation with the length of stay and mortality (Table 3).

Linear regression analysis for determining the association of patient mortality parameters with postoperative femur fracture in the ICU is shown in Table 4. The albumin level shows a strong negative association with mortality (B = −0.40, *p* = 0.001). Low albumin levels are associated with poor prognosis. D-dimer levels have a strong positive association with mortality (B = 0.45, *p* = 0.001). This suggests that increasing D-dimer levels increases the risk of mortality associated with thrombotic events. The CRP level also shows a strong positive association with mortality (B = 0.50, *p* = 0.001). CCI and APACHE II scores have a strong positive association with mortality (B = 0.55, *p* < 0.001 and B = 0.60, *p* < 0.001, respectively). Inflammatory markers such as PIV (B = 0.55, *p* < 0.001) and CAR (B = 0.50, *p* < 0.001) play a strong role in predicting mortality. NLR also has a significant positive association with mortality (B = 0.50, *p* < 0.001) (Table 4).

Multiple logistic regression analysis for determining the association of patient mortality parameters with postoperative femur fracture in the ICU is shown in Table 5. For every 1 g/dL reduction in albumin, the risk of ICU mortality increases 2.2-fold. Similarly, a 45% increase in mortality risk is observed with higher troponin levels, while a 40% increase in mortality risk is associated with elevated D-dimer levels. RDW also correlates with a 30% increased mortality risk. CRP is a strong marker in predicting mortality (OR = 1.50, *p* < 0.001). Procalcitonin levels are also positively associated with mortality (OR = 1.35, *p* = 0.002). Both CCI (OR = 1.25, *p* < 0.001) and APACHE II score (OR = 1.80, *p* < 0.001) are very strong predictors of mortality. Both PIV (OR = 1.80, *p* < 0.001) and CAR (OR = 1.75, *p* < 0.001) are positively associated with mortality. NLR (OR = 1.80, *p* < 0.001) and CLR (OR = 1.70, *p* < 0.001) are significant predictors of mortality. Prolonged ICU stay (>10 days) has a strong association with mortality (OR = 1.55, *p* < 0.001) (Table 5).

**Figure 1:**
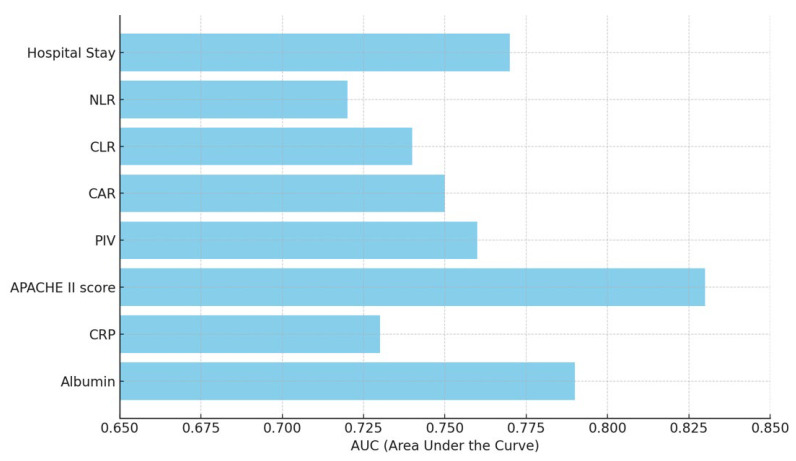
ROC analysis results of postoperative femur fracture mortality. PIV: Pan-Immune-Inflammation Value; CAR: CRP-to-ALB ratio; CLR: CRP-to-lymphocyte ratio; NLR: neutrophil-to-lymphocyte ratio.

The ROC analysis revealed that the APACHE II score, albumin levels, and hospital stay duration were the most effective predictors of mortality in postoperative femur fracture patients in the ICU. Among these, the APACHE II score demonstrated the highest predictive accuracy, with a cut-off point of >23, yielding an AUC of 0.83 (95% CI: 0.75–0.88), a sensitivity of 76%, and a specificity of 84% (*p* < 0.001). This highlights the significance of APACHE II as a robust clinical tool for mortality risk assessment. Albumin levels also proved to be a strong predictor, with a cut-off point of <2.5 g/dL, achieving an AUC of 0.79 (95% CI: 0.72–0.85). The sensitivity and specificity were 75% and 78%, respectively (*p* < 0.001), indicating the critical role of hypoalbuminemia as a marker of poor prognosis, likely reflecting malnutrition and systemic inflammation. Additionally, the duration of hospital stay emerged as a significant factor, with stays exceeding 10 days correlating with higher mortality. The ROC analysis showed an AUC of 0.77 (95% CI: 0.69–0.82), with a sensitivity of 74% and a specificity of 75% (*p* < 0.001). This finding underscores the association between prolonged ICU admission and increased risk of complications or adverse outcomes (Table 6, Figure 1).

## 4. Discussion

In this study, we evaluated the associations between inflammatory and metabolic markers and mortality in patients with postoperative femur fractures who were followed in the intensive care unit. Our findings suggest that the severity of the inflammatory response and nutritional status are correlated with mortality in these patients. While the observed associations were moderate, the statistically significant relationships identified for markers such as low albumin, high CRP, D-dimer, PIV, NLR, and CLR highlight their potential role in predicting adverse outcomes. These results align with the existing literature, underscoring the relevance of inflammatory and metabolic markers in postoperative risk stratification.

The albumin level stands out as an important parameter in predicting mortality. Albumin is a marker reflecting malnutrition and systemic inflammation in critically ill patients [5,6,7]. Niccolai et al. showed that low preoperative albumin and WBC levels associated with femoral neck fractures in elderly patients were found to be directly associated with poorer outcomes in these patients [14]. Hu et al. showed that low albumin levels increase mortality in intensive care patients [6]. Tie et al. also reported that low albumin levels are important in predicting postoperative complications and mortality [7]. Cirik et al. reported that low albumin levels and high CRP, procalcitonin, urea nitrogen, and creatinine levels were identified as other factors that increased the risk of mortality, while prolonged hospital stay was associated with survival [5]. In our study, hypoalbuminemia (<2.5 g/dL) demonstrated a strong association with ICU mortality, showing an AUC of 0.79 and a high predictive accuracy, with a sensitivity of 75% and specificity of 78%.

CRP is a marker of acute phase inflammation and has been found to be associated with mortality in various studies. Yamada et al. stated that CRP is used to predict mortality in early surgery in proximal femoral fractures patients and that CRP levels indicate the severity of the inflammatory response [15]. Similarly, Chen et al. reported that CRP reflects the severity of inflammation in critically ill patients and is associated with one-year mortality after hip fracture surgery for geriatric patients [22]. Hong et al. reported that CRP is a reliable marker of inflammation, with its elevated levels correlating with increased mortality risk in postoperative patients with femoral neck fracture [23]. Lin et al. also reported that CAR provides better prognostic information than CRP alone, especially in patients with underlying comorbidities like cancer and surgery [24]. In our study, CRP, with a cut-off value of 62.8 mg/L, had moderate sensitivity (52%) but high specificity (77%), yielding an AUC of 0.73 for mortality. The combination of CRP with other markers, such as in the CRP-to-ALB ratio (CAR), improved the predictive value, with CAR showing an AUC of 0.75 for mortality.

APACHE II is a clinical scoring system that evaluates the severity of disease in intensive care patients and predicts mortality. Vyhnanek et al. stated that the APACHE II score is a reliable tool for predicting the risk of mortality trauma care in the geriatric population [25]. Widyastuti et al. showed that the APACHE II score is especially effective in predicting mortality in orthopedic intensive care patients [26]. Cirik et al. studied the prognostic factors affecting 30-day mortality in geriatric patients over 65 years of age admitted to the intensive care unit with acute respiratory failure. They found that higher APACHE II score, Charlson Comorbidity Index, and need for inotropic support were significantly associated with mortality [5]. In our study, we found that a high APACHE II score is strongly associated with mortality. The APACHE II score (>23) demonstrated excellent predictive capability in our study, with an AUC of 0.83, a sensitivity of 76%, and a specificity of 84%.

There are also limited studies showing the association of inflammatory markers such as PIV, CAR, CLR, and NLR in predicting mortality [24,27,28,29]. Huang et al. reported that PIV is a valuable prognostic tool for critically ill patients, particularly in predicting outcomes in sepsis and systemic inflammation [30]. Cakin et al. reported that NLR is a reliable parameter in predicting mortality by reflecting the balance between inflammation and immune response [27]. Ntalouka et al. showed that the combination scores of NLR and CLR, based on admission values, are promising predictors for mortality in patients suffering from severe COVID-19 infection [19]. In our study, PIV, which integrates immune cell counts and inflammatory markers, showed strong predictive power in our study, with an AUC of 0.76. Similarly, both NLR and CLR showed significant associations with mortality, with AUC values of 0.72 and 0.74, respectively.

In our study, prolonged hospital stays (>10 days) were associated with increased mortality risk, with an AUC of 0.77, a sensitivity of 74%, and a specificity of 75%. This finding is supported by Darden et al., who found that prolonged ICU stays are correlated with higher rates of mortality due to nosocomial infections, increased inflammatory responses, and the development of multiple-organ dysfunction after surgery [31]. Our study further validates the role of prolonged hospitalization as a marker of poor prognosis in postoperative ICU patients, aligning with the work of Lee et al., who highlighted that length of stay in the ICU is a critical determinant of overall patient outcomes [32].

### Limitations of the Study

This study has some limitations. First, since the study was designed retrospectively, there may be missing data or information loss. In addition, the fact that the study was conducted in a single center limits the generalizability of the results. The relatively low number of patients is also a factor that limits statistical power. Multicenter prospective studies should be conducted in larger patient groups.

One of the strengths of this study is that both classical clinical scores and new inflammatory biomarkers were evaluated together in intensive care patients with postoperative femur fractures. In our study, the use of common clinical scores such as APACHE II and the Charlson Comorbidity Index, as well as new biomarkers such as PIV, CAR, and CLR, offers a unique approach to predicting mortality. In addition, this study addresses a wide range of factors affecting mortality in intensive care patients after femur fractures in the literature, emphasizing the importance of inflammation and nutritional status.

## 5. Conclusions

This study successfully demonstrates the utility of combining traditional clinical markers, such as the APACHE II score, with novel inflammatory markers, such as PIV, CAR, and NLR, in predicting mortality in ICU patients following femur fracture surgery. The integration of emerging biomarkers with well-established scoring systems offers enhanced predictive accuracy and provides valuable insights into patient management. The study contributes significantly to the growing body of literature focused on risk stratification in critically ill orthopedic patients. Future research should focus on expanding these findings through larger, multi-center studies and exploring the application of these biomarkers in other high-risk surgical populations, including patients undergoing major abdominal or cardiovascular surgeries. Additionally, longitudinal analysis of biomarker trends during ICU stays could provide deeper insights into their dynamic changes and predictive value over time, potentially guiding more personalized and time-sensitive interventions.

## Figures and Tables

**Table 1:** Sociodemographic and laboratory findings of patients with postoperative femur fracture in the ICU.

	Patients (N = 121) Mean ± SD/Frequency (%)
Age	76.3 ± 9.6 years
Gender	Male: 58 (47.9%); female: 63 (52.1%)
Diabetes, n (%)	60 (49.6%)
Hypertension, n (%)	82 (67.8%)
CAD, n (%)	35 (28.9%)
Heart failure, n (%)	24 (19.8%)
CKD, n (%)	17 (14.0%)
COPD, n (%)	7 (5.8%)
Malignancy, n (%)	8 (6.6%)
WBC (×10^3^/µL)	11.2 ± 3.5
RDW (%)	15.5 ± 2.0
Platelet (Plt) (×10^3^/µL)	230 ± 50
Neutrophil (×10^3^/µL)	7.0 ± 2.2
MPV (fL)	9.5 ± 1.0
Monocyte (×10^3^/µL)	0.7 ± 0.2
Lymphocyte (×10^3^/µL)	1.5 ± 0.6
Ca++ (mg/dL)	9.0 ± 0.5
Glucose (mg/dL)	120 ± 30
Albumin (g/dL)	3.5 ± 0.5
BUN (mmol/L)	15 ± 5
Creatinine (mg/dL)	1.1 ± 0.3
Troponin (U/L)	0.05 ± 0.02
D-dimer (mg/L)	1.5 ± 0.5
CRP (mg/L)	50 ± 20
Procalcitonin (ng/L)	0.5 ± 0.3
CCI	4.5 ± 1.5
APACHE II score	18 ± 5
PIV	406.50 ± 102.50
CAR	1.5 ± 0.5
CLR	5.5 ± 2.3
PLR	180 ± 45
NLR	4.5 ± 1.8
ICU Stay (days)	8.2 ± 5.6
ICU Mortality	24 (20%)

**Table 2:** Comparison of parameters of survival and mortality of patients with postoperative femur fracture in the ICU.

Parameters	Survivor (N = 97) Mean ± SD, Frequency (%)	Mortality (N = 24) Mean ± SD, Frequency (%)	*p* Value
Diabetes, n (%)	48 (49.5%)	12 (50%)	0.88
Hypertension, n (%)	68 (70.1%)	14 (58.3%)	0.22
CAD, n (%)	32 (33%)	8 (33.3%)	0.95
Heart failure, n (%)	18 (18.5%)	6 (25%)	0.42
CKD, n (%)	15 (15.5%)	5 (20.8%)	0.55
COPD, n (%)	5 (5.1%)	2 (8.3%)	0.52
Malignancy, n (%)	6 (6.2%)	2 (8.3%)	0.72
WBC (×10^3^/µL)	10.8 ± 3.2	12.2 ± 3.7	0.03 *
RDW (%)	15.2 ± 1.9	16.3 ± 2.1	0.04 *
Platelet (Plt) (×10^3^/µL)	235 ± 45	220 ± 38	0.07
Neutrophil (×10^3^/µL)	6.8 ± 2.0	7.5 ± 2.5	0.08
MPV (fL)	9.4 ± 0.9	9.8 ± 1.1	0.15
Monocyte (×10^3^/µL)	0.65 ± 0.15	0.73 ± 0.18	0.12
Lymphocyte (×10^3^/µL)	1.6 ± 0.6	1.3 ± 0.5	0.04 *
Ca++ (mg/dL)	9.1 ± 0.4	8.8 ± 0.5	0.02 *
Glucose (mg/dL)	118 ± 27	130 ± 35	0.09
Albumin (g/dL) (ALB)	3.7 ± 0.4	3.2 ± 0.6	0.001 **
BUN (mmol/L)	14.8 ± 4.8	17.5 ± 6.5	0.05
Creatinine (mg/dL)	1.0 ± 0.25	1.2 ± 0.4	0.02 *
Troponin (U/L)	0.04 ± 0.02	0.08 ± 0.03	0.01 *
D-dimer (mg/L)	1.4 ± 0.6	2.1 ± 0.9	0.001 **
CRP (mg/L)	48 ± 19	70 ± 28	0.02 *
Procalcitonin (ng/L)	0.4 ± 0.2	0.7 ± 0.3	0.03 *
CCI	4.2 ± 1.4	5.6 ± 1.2	0.04 *
APACHE II score	14 ± 4	26 ± 2	0.001 **
PIV	350 ± 120	470 ± 140	0.03 *
CAR	1.4 ± 0.6	2.1 ± 0.8	0.02 *
CLR	4.8 ± 1.8	6.5 ± 2.3	0.04 *
PLR	170 ± 50	200 ± 45	0.04 *
NLR	4.2 ± 1.5	5.6 ± 2.1	0.02 *
Hospital stay (days)	7.5 ± 4.3	11.5 ± 5.1	0.001 **

* *p* < 0.05, ** *p* < 0.01.

**Table 3:** The correlation between the length of hospital stay and mortality of patients with postoperative femur fracture in the ICU.

Parameters	Hospital Stay (Days) r Value, *p* Value	Mortality r Value, *p* Value
WBC (×10^3^/µL)	r = 0.32, *p* = 0.01	r = 0.35, *p* = 0.01
RDW (%)	r = 0.45, *p* = 0.002	r = 0.47, *p* = 0.001
Platelet (Plt) (×10^3^/µL)	r = −0.24, *p* = 0.03	r = −0.20, *p* = 0.06
Neutrophil (×10^3^/µL)	r = 0.38, *p* = 0.003	r = 0.41, *p* = 0.002
MPV (fL)	r = 0.28, *p* = 0.02	r = 0.33, *p* = 0.01
Monocyte (×10^3^/µL)	r = 0.22, *p* = 0.05	r = 0.20, *p* = 0.07
Lymphocyte (×10^3^/µL)	r = −0.30, *p* = 0.01	r = −0.35, *p* = 0.01
Ca++ (mg/dL)	r = −0.27, *p* = 0.03	r = −0.32, *p* = 0.01
Glucose (mg/dL)	r = 0.33, *p* = 0.01	r = 0.37, *p* = 0.01
Albumin (g/dL) (ALB)	r = −0.48, *p* < 0.001	r = -0.52, *p* < 0.001
BUN (mmol/L)	r = 0.39, *p* = 0.002	r = 0.42, *p* = 0.001
Creatinine (mg/dL)	r = 0.36, *p* = 0.01	r = 0.39, *p* = 0.01
Troponin (U/L)	r = 0.42, *p* = 0.001	r = 0.47, *p* < 0.001
D-dimer (mg/L)	r = 0.40, *p* = 0.002	r = 0.46, *p* = 0.001
CRP (mg/L)	r = 0.49, *p* < 0.001	r = 0.53, *p* < 0.001
Procalcitonin (ng/L)	r = 0.46, *p* = 0.001	r = 0.50, *p* < 0.001
CCI	r = 0.50, *p* < 0.001	r = 0.55, *p* < 0.001
APACHE II score	r = 0.54, *p* < 0.001	r = 0.60, *p* < 0.001
PIV	r = 0.41, *p* = 0.002	r = 0.48, *p* = 0.001
CAR	r = 0.45, *p* = 0.001	r = 0.51, *p* < 0.001
CLR	r = 0.42, *p* = 0.001	r = 0.48, *p* = 0.001
PLR	r = 0.30, *p* = 0.02	r = 0.35, *p* = 0.01
NLR	r = 0.48, *p* < 0.001	r = 0.53, *p* < 0.001

**Table 4:** Linear regression analysis for determining the association of patient mortality parameters with postoperative femur fracture in the ICU.

Parameters	B	St. Error	Beta	t	*p*-Value
WBC (×10^3^/µL)	0.25	0.12	0.20	2.08	0.04 *
RDW (%)	0.35	0.10	0.30	3.50	0.001 **
Platelet (Plt) (×10^3^/µL)	−0.18	0.09	−0.15	−2.00	0.05 *
Neutrophil (×10^3^/µL)	0.28	0.11	0.22	2.45	0.02 *
MPV (fL)	0.20	0.10	0.18	2.00	0.05 *
Monocyte (×10^3^/µL)	0.12	0.08	0.10	1.50	0.14
Lymphocyte (×10^3^/µL)	−0.25	0.09	−0.20	−2.78	0.01 **
Ca++ (mg/dL)	−0.22	0.08	−0.25	−2.75	0.01 **
Glucose (mg/dL)	0.15	0.09	0.14	1.67	0.09
Albumin (g/dL)	−0.40	0.12	−0.35	−3.33	0.001 **
BUN (mmol/L)	0.30	0.11	0.25	2.73	0.01 **
Creatinine (mg/dL)	0.20	0.10	0.18	2.00	0.05 *
Troponin (U/L)	0.35	0.12	0.30	2.92	0.01 **
D-dimer (mg/L)	0.45	0.13	0.40	3.46	0.001 **
CRP (mg/L)	0.50	0.15	0.45	3.33	0.001 **
Procalcitonin (ng/L)	0.30	0.11	0.25	2.73	0.01 **
CCI	0.55	0.14	0.50	3.93	<0.001 **
APACHE II score	0.60	0.15	0.55	4.00	<0.001 **
PIV	0.55	0.13	0.50	4.23	<0.001 **
CAR	0.50	0.12	0.45	4.17	<0.001 **
CLR	0.48	0.11	0.44	4.10	<0.001 **
PLR	0.35	0.10	0.30	3.50	0.001 **
NLR	0.50	0.13	0.45	3.85	<0.001 **
Hospital stay (days)	0.55	0.14	0.50	3.93	<0.001 **

* *p* < 0.05, ** *p* < 0.01.

**Table 5:** Multiple logistic regression analysis for determining the association of patient mortality parameters with postoperative femur fracture in the ICU.

	ICU Mortality		
	Odds Ratio	95% CI	*p* Value
Diabetes	−1.10	0.85–1.40	0.22
Hypertension	−1.15	0.90–1.35	0.18
RDW	+1.30	1.12–1.48	0.002 **
Albumin	−2.20	1.60–3.00	<0.001 **
Troponin	+1.45	1.20–1.70	<0.001 **
D-dimer	+1.40	1.15–1.65	0.001 **
CRP	+1.50	1.35–1.75	<0.001 **
Procalcitonin	+1.35	1.15–1.55	0.002 **
CCI	+1.25	1.10–1.40	<0.001 **
APACHE II score	+1.80	1.50–2.00	<0.001 **
PIV	+1.80	1.50–2.20	<0.001 **
CAR	+1.75	1.45–2.15	<0.001 **
CLR	+1.70	1.45–2.00	<0.001 **
PLR	+1.40	1.10–1.70	0.01 *
NLR	+1.80	1.55–2.15	<0.001 **
Hospital stay (days)	+1.55	1.30–1.80	<0.001 **

* *p* < 0.05, ** *p* < 0.01.

**Table 6:** ROC analysis results of postoperative femur fracture mortality.

	Cut-Off	Sensitivity	Specificity	AUC (95% CI)	*p* Value
Albumin	<2.5 g/dL	75%	78%	0.79 (0.72–0.85)	<0.001 **
CRP	>62.8 mg/L	52%	77%	0.73 (0.65–0.81)	0.001 **
APACHE II score	>23	76%	84%	0.83 (0.75–0.88)	<0.001 **
PIV	>450	70%	72%	0.76 (0.68–0.82)	<0.001 **
CAR	>2.94	66%	73%	0.75 (0.68–0.80)	<0.001 **
CLR	>14.0	64%	71%	0.74 (0.67–0.79)	<0.001 **
NLR	>10.0	60%	70%	0.72 (0.65–0.77)	<0.001 **
Hospital stay	>10 days	74%	75%	0.77 (0.69–0.82)	<0.001 **

CRP: C-reactive protein; PIV: Pan-Immune-Inflammation Value; CAR: CRP-to-ALB ratio; CLR: CRP-to-lymphocyte ratio; NLR: neutrophil-to-lymphocyte ratio. **: *p* < 0.01.

## Data Availability

The original contributions presented in the study are included in the article; further inquiries can be directed to the corresponding authors.

## References

[1] Halvachizadeh S., Martin D.P., Pfeifer R., Jukema G.N., Gueorguiev B., Pape H.C., Berk T. (2023). Which non-infection related risk factors are associated with impaired proximal femur fracture healing in patients under the age of 70 years?. BMC Musculoskelet. Disord..

[2] Becker N., Hafner T., Pishnamaz M., Hildebrand F., Kobbe P. (2022). Patient-specific risk factors for adverse outcomes following geriatric proximal femur fractures. Eur. J. Trauma Emerg. Surg..

[3] Barbosa T.A., Souza A.M.F., Leme F.C.O., Grassi L.D.V., Cintra F.B., Lima R.M.E., Gumieiro D.N., Lima L. (2019). Perioperative complications and mortality in elderly patients following surgery for femoral fracture: Prospective observational study. Braz. J. Anesthesiol..

[4] Merchan-Galvis A.M., Munoz-Garcia D.A., Solano F., Velasquez J.C., Sotelo N.F., Molina D.A., Caicedo J.P., Concha J.M., Calvache J.A., Martinez-Zapata M.J. (2023). Delayed surgery and health related quality of life in patients with proximal femoral fracture. Sci. Rep..

[5] Cirik M.Ö., Yenibertiz D. (2020). What are the prognostic factors affecting 30-day mortality in geriatric patients with respiratory failure in the Intensive Care Unit?. Pak. J. Med. Sci..

[6] Hu Z., Song C., Zhang J. (2024). Elevated serum albumin-to-creatinine ratio as a protective factor on clinical outcomes among critically ill patients with sepsis: A retrospective study. Front. Med..

[7] Tie X., Zhao Y., Sun T., Zhou R., Li J., Su J., Yin W. (2024). Associations between serum albumin level trajectories and clinical outcomes in sepsis patients in ICU: Insights from longitudinal group trajectory modeling. Front. Nutr..

[8] Skouras A.Z., Antonakis-Karamintzas D., Tsolakis C., Tsantes A.E., Kourlaba G., Zafeiris I., Soucacos F., Papagiannis G., Triantafyllou A., Houhoula D. (2023). Pre- and Postoperative Exercise Effectiveness in Mobility, Hemostatic Balance, and Prognostic Biomarkers in Hip Fracture Patients: A Study Protocol for a Randomized Controlled Trial. Biomedicines.

[9] Tschon M., Contartese D., Pagani S., Borsari V., Fini M. (2021). Gender and Sex Are Key Determinants in Osteoarthritis Not Only Confounding Variables. A Systematic Review of Clinical Data. J. Clin. Med..

[10] Ko D.E., Yoon H.J., Nam S.B., Song S.W., Lee G., Ham S.Y. (2021). Preoperative Neutrophil to Lymphocyte Ratio, Platelet to Lymphocyte Ratio, and Mean Platelet Volume as Predictors of 1-Year Mortality in Patients Undergoing an Open Repair of Abdominal Aortic Aneurysms: A Retrospective Study. J. Clin. Med..

[11] Alsabani M.H., Alenezi F.K., Alotaibi B.A., Alotaibi A.A., Olayan L.H., Aljurais S.F., Alarfaj N., Alkhurbush D., Almuhaisen G., Alkhmies L. (2024). Ratios of Neutrophils and Platelets to Lymphocytes as Predictors of Postoperative Intensive Care Unit Admission and Length of Stay in Bariatric Surgery Patients: A Retrospective Study. Medicina.

[12] Sefil F., Ulutas K.T., Dokuyucu R., Sumbul A.T., Yengil E., Yagiz A.E., Yula E., Ustun I., Gokce C. (2014). Investigation of neutrophil lymphocyte ratio and blood glucose regulation in patients with type 2 diabetes mellitus. J. Int. Med. Res..

[13] Chen Y., Tu C., Liu G., Peng W., Zhang J., Ge Y., Tan Z., Bei M., Gao F., Tian M. (2024). Association between admission inflammatory indicators and 3-year mortality risk in geriatric patients after hip fracture surgery: A retrospective cohort study. Front. Surg..

[14] Niccolai F., Parchi P.D., Vigorito A., Pasqualetti G., Monzani F., Lisanti M. (2016). The correlation between preoperative levels of albumin and tlc and mortality in patients with femoral neck fracture. J. Biol. Regul. Homeost. Agents.

[15] Yamada Y., Kotani T., Kishida S., Ogata Y., Okuwaki S., Ohyama S., Iwata S., Iijima Y., Ise S., Sakuma T. (2024). Factors influencing the achievement of early surgery in proximal femoral fractures under a Japanese incentive policy. J. Orthop. Sci..

[16] Bilgin M., Akkaya E., Dokuyucu R. (2024). Inflammatory and Metabolic Predictors of Mortality in Pulmonary Thromboembolism: A Focus on the Triglyceride–Glucose Index and Pan-Immune Inflammation Value. J. Clin. Med..

[17] Bilgin M., Akkaya E., Dokuyucu R. (2024). The Role of Triglyceride/HDL Ratio, Triglyceride–Glucose Index, and Pan-Immune-Inflammation Value in the Differential Diagnosis of Acute Coronary Syndrome and Predicting Mortality. J. Clin. Med..

[18] Pinerua-Gonsalvez J.F., Ruiz Rebollo M.L., Zambrano-Infantino R.D.C., Rizzo-Rodriguez M.A., Fernandez-Salazar L. (2023). Value of CRP/albumin ratio as a prognostic marker of acute pancreatitis: A retrospective study. Rev. Esp. Enferm. Dig..

[19] Ntalouka M.P., Brotis A., Mermiri M., Pagonis A., Chatzis A., Bareka M., Kotsi P., Pantazopoulos I., Gourgoulianis K., Arnaoutoglou E.M. (2024). Predicting the Outcome of Patients with Severe COVID-19 with Simple Inflammatory Biomarkers: The Utility of Novel Combined Scores-Results from a European Tertiary/Referral Centre. J. Clin. Med..

[20] Qu R., Hu L., Ling Y., Hou Y., Fang H., Zhang H., Liang S., He Z., Fang M., Li J. (2020). C-reactive protein concentration as a risk predictor of mortality in intensive care unit: A multicenter, prospective, observational study. BMC Anesthesiol..

[21] Segmen F., Aydemir S., Kucuk O., Dokuyucu R. (2024). The Roles of Vitamin D Levels, Gla-Rich Protein (GRP) and Matrix Gla Protein (MGP), and Inflammatory Markers in Predicting Mortality in Intensive Care Patients: A New Biomarker Link?. Metabolites.

[22] Chen Y., Guo Y., Tong G., He Y., Zhang R., Liu Q. (2024). Combined nutritional status and activities of daily living disability is associated with one-year mortality after hip fracture surgery for geriatric patients: A retrospective cohort study. Aging Clin. Exp. Res..

[23] Xu H., Xie J.-W., Liu L., Wang D., Huang Z.-Y., Zhou Z.-K. (2021). Combination of CRP with NLR is a sensitive tool for screening fixation-related infection in patients undergoing conversion total hip arthroplasty after failed internal fixation for femoral neck fracture. Bone Jt. J..

[24] Lin J., Liang H., Zheng H., Li S., Liu H., Ge X. (2023). CONUT can be a predictor of postoperative complications after laparoscopic-assisted radical gastrectomy for elderly gastric cancer patients. Medicine.

[25] Vyhnanek F., Ocadlik M., Drienko M., Gurlich R. (2020). Trauma Care on Geriatric Population in Trauma Centre Faculty Hospital Kralovske Vinohrady in Prague. Cas. Lek. Cesk.

[26] Widyastuti Y., Zaki W.A., Widodo U., Jufan A.Y., Pratomo B.Y. (2022). Predictive accuracy of the APACHE IV scores on mortality and prolonged stay in the intensive care unit of Dr Sardjito Hospital. Med. J. Malays..

[27] Cakin O., Karaveli A., Yuce Aktepe M., Gumus A., Yildirim O.E. (2024). Comparison of Inflammatory Marker Scoring Systems and Conventional Inflammatory Markers in Patients over 65 Years of Age Admitted to the Intensive Care Unit: A Multicenter, Retrospective, Cohort Study. J. Clin. Med..

[28] Shimoda M., Udo R., Imasato R., Oshiro Y., Suzuki S. (2021). What are the risk factors of conversion from total cholecystectomy to bailout surgery?. Surg. Endosc..

[29] Xu H.B., Xu Y.H., He Y., Lin X.H., Suo Z., Shu H., Zhang H. (2024). Association between admission pan-immune-inflammation value and short-term mortality in septic patients: A retrospective cohort study. Sci. Rep..

[30] Huang Y.W., Zhang Y., Li Z.P., Yin X.S. (2023). Association between a four-parameter inflammatory index and all-cause mortality in critical ill patients with non-traumatic subarachnoid hemorrhage: A retrospective analysis of the MIMIC-IV database (2012–2019). Front. Immunol..

[31] Darden D.B., Brakenridge S.C., Efron P.A., Ghita G.L., Fenner B.P., Kelly L.S., Mohr A.M., Moldawer L.L., Moore F.A. (2021). Biomarker Evidence of the Persistent Inflammation, Immunosuppression and Catabolism Syndrome (PICS) in Chronic Critical Illness (CCI) After Surgical Sepsis. Ann. Surg..

[32] Lee K.S., Min H.S., Moon J.Y., Lim D., Kim Y., Ko E., Kim Y.S., Kim J., Lee J., Sung H.K. (2022). Patient and hospital characteristics predict prolonged emergency department length of stay and in-hospital mortality: A nationwide analysis in Korea. BMC Emerg. Med..

